# The Structure of Metal Binding Domain 1 of the Copper Transporter ATP7B Reveals Mechanism of a Singular Wilson Disease Mutation

**DOI:** 10.1038/s41598-017-18951-1

**Published:** 2018-01-12

**Authors:** Corey H. Yu, Woonghee Lee, Sergiy Nokhrin, Oleg Y. Dmitriev

**Affiliations:** 1 Department of Biochemistry, University of Saskatchewan, Saskatoon, SK Canada; 2 NMRFAM, University of Wisconsin, Madison, WI USA

## Abstract

Copper-transporter ATP7B maintains copper homeostasis in the human cells and delivers copper to the biosynthetic pathways for incorporation into the newly synthesized copper-containing proteins. ATP7B is a target of several hundred mutations that lead to Wilson disease, a chronic copper toxicosis. ATP7B contains a chain of six cytosolic metal-binding domains (MBDs), the first four of which (MBD1-4) are believed to be regulatory, and the last two (MBD5-6) are required for enzyme activity. We report the NMR structure of MBD1, the last unsolved metal-binding domain of ATP7B. The structure reveals the disruptive mechanism of G85V mutation, one of the very few disease causing missense mutations in the MBD1-4 region of ATP7B.

## Introduction

Membrane transporter ATP7B regulates the level and intracellular distribution of copper in human tissues^1^. At the basal copper levels, ATP7B is located in the *trans*-Golgi network, where it delivers copper to the newly synthesized copper-containing proteins, such as ceruloplasmin. At the elevated copper levels, ATP7B relocates to the cytosolic membrane vesicles and plasma membrane, where it exports copper from the cell. This function is particularly important in the liver, where copper excretion into the bile serves as a main route of excess copper disposal in the human body.

**Figure 1:**
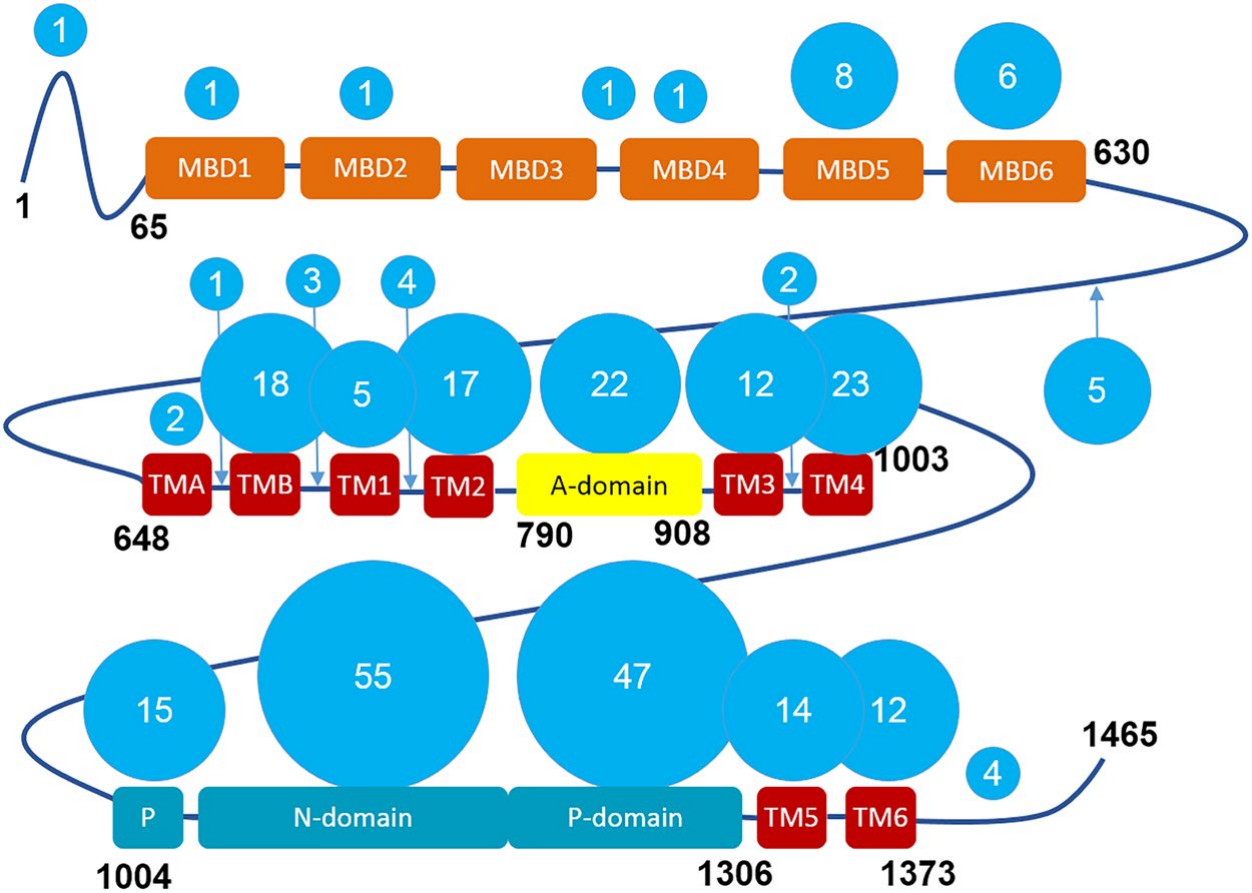
Domain composition of ATP7B and the distribution of the Wilson disease mutations. ATP7B includes six cytosolic metal binding domains (MBD1-MBD6, *orange*), eight transmembrane helices (TMA-TM6, *red*), and the nucleotide-binding (N) and phosphorylation (P) domains (*cyan*), which together hydrolyze ATP, with the participation of the actuator (A) domain (*yellow*). The length of the interdomain linkers is not to scale. The number of known Wilson disease causing missense mutations in each domain and in the connecting loops, defined as distinct single amino acid substitutions, is shown in the blue circles. The list of mutations, as of 2010, was obtained from the Wilson disease mutation database (http://www.wilsondisease.med.ualberta.ca/database.asp)^43^. Except for the metal binding domains, a homology model of ATP7B^5^ based on the X-ray structure of the bacterial copper ATPase CopA^2^ was used to confirm domain assignment of the mutation sites.

ATP7B uses the energy of ATP hydrolysis to translocate copper across the membrane. It belongs to the large family of P-type ATPases, and shares much of the domain composition and some fundamental features of the catalytic mechanism with the better studied members of the family, such as Ca-ATPase and Na,K-ATPase^1–7^. There is no high-resolution structure of ATP7B, but the structures of most cytosolic domains have been solved by NMR, and the overall structure of ATP7B has been modeled by homology^5^ using the X-ray structure of the bacterial copper ATPase CopA from *Legionella pneumophila*^2^ as a template. This model does not include the N-terminal chain of the six cytosolic metal-binding domains (MBDs) connected by flexible loops of various length, a unique structural feature of ATP7B and of the closely related copper transporter ATP7A (Fig. 1).

**Figure 2:**
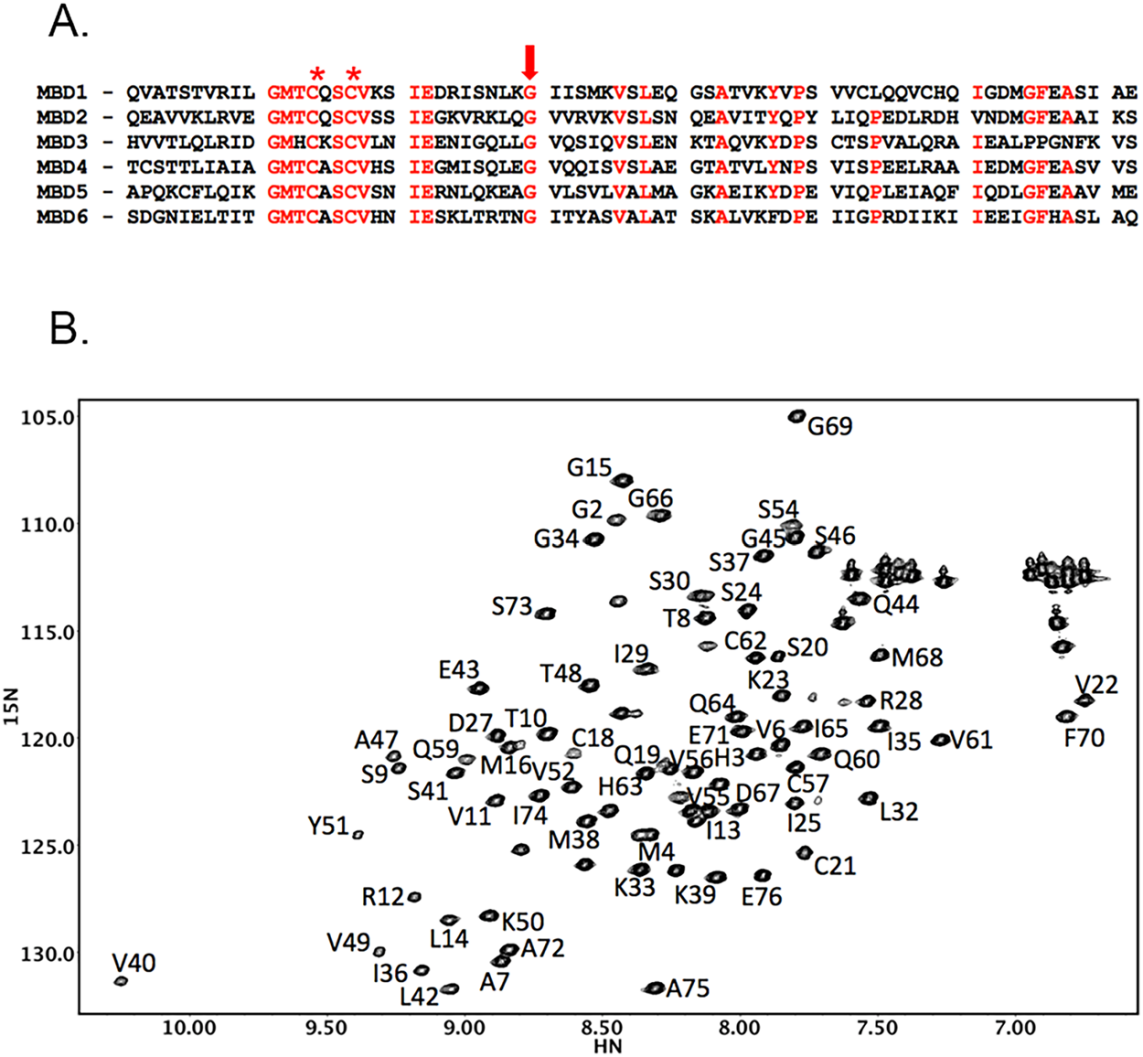
Amino acid sequence of the metal binding domains of ATP7B (**A**) and a fingerprint 1H,15N-HSQC spectrum of MBD1 (**B**). (**A**) Conserved residues are shown in red. Cysteine residues in the copper binding motif of the MBDs are marked by an asterisk. The invariant glycine, which is a target of the Wilson disease causing mutation in MBD1 (G85V), is shown by the arrow. (**B**) The sequential amino acid assignments in MBD1 are shown. In the protein construct used for structure determination, residues 1–4 are from the purification tag, and Q5 corresponds to Q56 in the full length ATP7B.

The structures of ATP7B metal-binding domains 2–6 have been solved previously by NMR^8–10^. Each of the six MBDs has a conserved ferredoxin-like fold and is approximately 70 amino acids long. The individual MBDs show significant sequence homology to each other, with the invariant GM(T/H)CxSCxxxIE motif responsible for binding copper(I) ions^11,12^ through the sulfur atoms of the two cysteine residues (Fig. 2A). Copper binding causes some changes in the local dynamics of the binding site, but does not alter overall conformation of the metal-binding domains^13–16^. Previous NMR studies show that MBD1-6 chain does not fold together into a compact structure, and the individual domains are highly mobile^17–19^.

In the cell, ATP7B receives copper from a chaperone protein Atox1, which is structurally rather similar to the MBDs^20,21^. Presumably, copper is transferred from Atox1 to some or all of the MBDs, then to the copper-binding site in the transmembrane domain, and, finally, to an acceptor on the other side of the membrane. However, the exact path of copper transfer is unknown. MBDs 5–6, which are closest to the membrane, are required for activity, while MBD1-4 are believed to play a regulatory role^22–24^.

Mutations that impair transport activity or disrupt intracellular targeting of ATP7B cause Wilson disease, chronic copper toxicosis that primarily affects the liver and the brain. Wilson disease is an autosomal recessive disorder with highly variable symptoms, onset age and progression. This variability stems from the diverse effects of several hundred known disease mutations, further complicated by the fact that most Wilson disease patients are compound heterozygotes. While molecular basis of many Wilson disease mutations is known, disruptive effect of others is still unexplained, and, generally, there is no reliable correlation between the mutation type and disease symptoms and prognosis.

Analysis of the domain distribution of Wilson disease missense mutations reveals a striking difference between the first four metal binding domains, and the rest of the protein. Out of about 300 single amino acid substitutions known to be associated with Wilson disease, only five are located in the MBD1-4 region, and only one of those five, G85V, in MBD1 (Fig. 1). By comparison, MBD5 and MBD6, both very similar in size and structure to MBDs 1–4, are targets of eight and six disease causing missense mutations respectively, while the N-domain, which binds ATP, is a target of 55 such mutations. The paucity of missense disease mutations in MBD1-4 may reflect the fact that these domains play a regulatory role, and, unlike MBDs 5-6, are not strictly required for copper transport activity. The molecular basis of the few known mutations in MBD1-4 is therefore particularly interesting and may offer a clue to the individual functional roles of these domains in the native enzyme. To determine the disruptive mechanism of the G85V mutation, we have solved the structure of MBD1, the last unsolved metal-binding domain of ATP7B.

## Results and Discussion

### Structure of MBD1

The protein construct used for MBD1 structure determination produced high quality spectra. Using standard triple resonance experiments, we assigned backbone amides of all non-proline residues (Fig. 2B), with the exception of Q5, T17, and L58. These residues are located in flexible regions of the protein, and the backbone amide signals may not be observable due to the chemical exchange. The structure was calculated from 628 NOE restraints, 119 backbone dihedral angle constraints, with 40 hydrogen bond restraints added during structure refinement. The final structure ensemble had an RMSD of 0.59 Å for the backbone atoms, and 1.15 Å for all heavy atoms (Supplementary Table S1).

**Figure 3:**
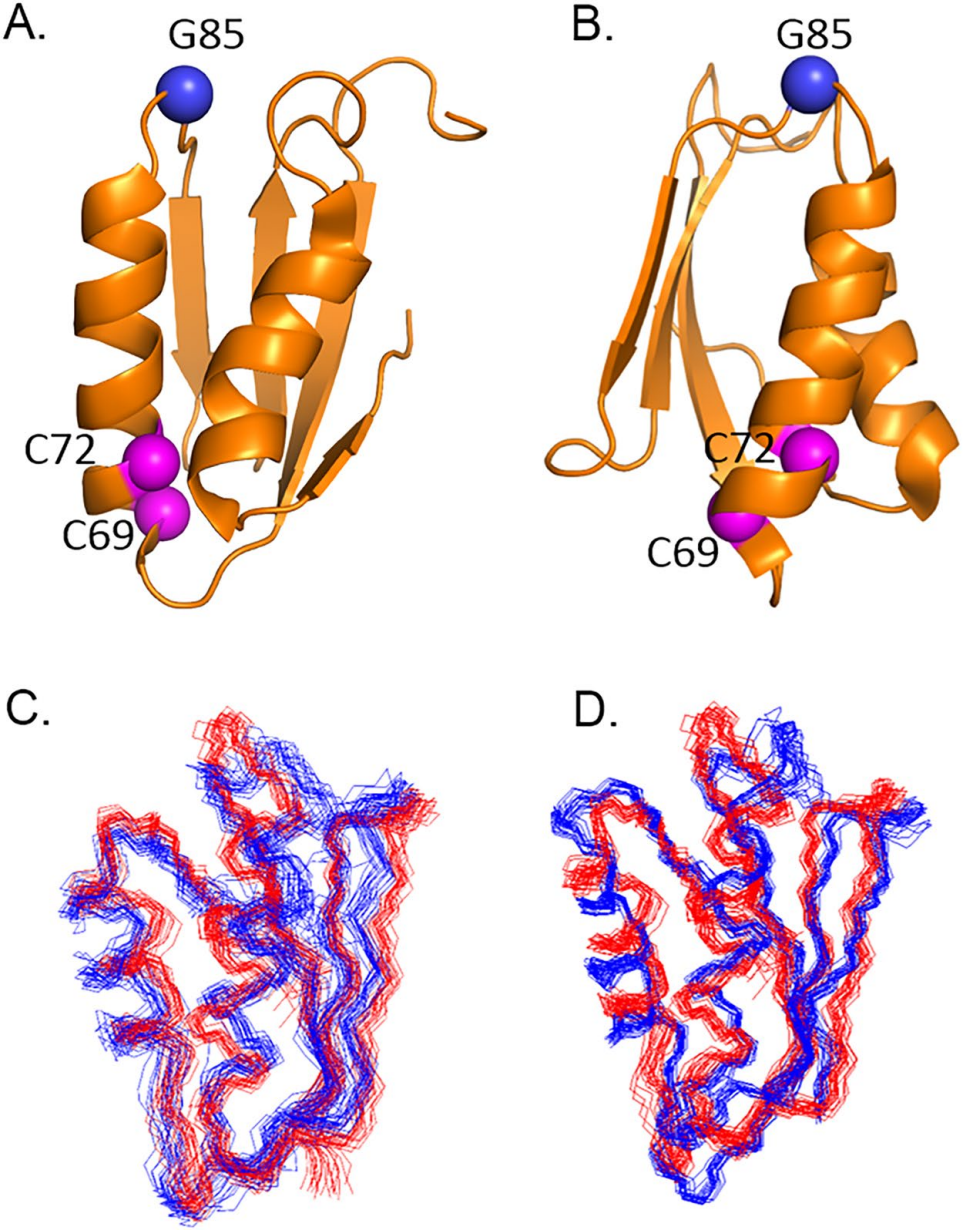
The structure of MBD1 of ATP7B. (**A**,**B**) The ribbon diagram of MBD1 structure with the invariant cysteines in the copper binding motif (C69 and C72 in the full-length protein, magenta), and G85 (blue) shown as spheres. Panels A and B are related by 90o rotation around the vertical axis. (**C**,**D**) MBD1 structure ensemble (PDB ID 2N7Y, red) aligned with the MBD6 (PDB ID 2EW9, C, blue) and MBD3 (PDB ID 2ROP, D, blue) ensembles by minimizing RMSD for the backbone atoms.

The MBD1 structure (Fig. 3A,B) shows the characteristic βαββαβ ferredoxin fold, similar to the other metal-binding domains of ATP7B, with the ensemble RMSD between the structures for the ordered core varying from 1.9 Å (MBD1 vs. MBD6, Fig. 3C) to 3.2 Å (MBD1 vs. MBD3, Fig. 3D). By comparison, the ensemble RMSD from MBD1 of ATP7A is 2.1 Å. The MBD1 rotational correlation time τ_c_ is 4.8 ns, as compared to 4.6 ns for MBD2^25^, for example, and is consistent with the monomeric state of the protein. Similar to the other MBDs, the copper binding motif CxxC appears to experience complex dynamics (Fig. S1), while the rest of the folded core of the protein is well ordered, with some flexibility in some of the connecting loop regions.

### Structural effect of the G85V mutation

The structure of MBD1 explained the effect of G85V mutation, one of the very few known missense Wilson disease causing mutation in the MBD1-4 region of the protein. Although not among the most frequent, the G85V mutation has been found in various ethnic populations^26–28^. Biochemically, the G85V variant of ATP7B showed reduced interaction with the copper chaperone Atox1^29^, but increased binding to COMMD1, a regulatory protein, involved in copper metabolism, among other cellular processes^30^. Previously, G85V substitution was also shown to cause the loss of ATP7B transport activity^31^, ATP7B retention in the endoplasmic reticulum, and rapid protein degradation^30^, indicative of protein misfolding. We were unable to purify G85V-MBD1 using the standard MBD purification procedure, presumably due to the degradation of the fusion protein in the bacterial cell. Taken together, these facts suggest that G85V mutation causes MBD1 misfolding.

The G85 residue is located in the loop connecting α1-helix and β2-strand. Interestingly, glycine in this position is conserved among all the MBDs of ATP7B (Fig. 2A), and, with one exception, all the MBDs of ATP7A. Yet, to date, only mutations of this glycine in MBD1 (G85V) and MBD6 (G591D) are known to cause Wilson disease. Analysis of the MBD local structure suggests an explanation of this distinct effect of the G85V mutation (Table 1).

**Table 1:** Backbone conformation of the conserved glycine residue in the ATP7B metal binding domains (MBDs).

MBD	PDB ID	Residue	φ	ψ	Wilson disease mutation
1	2N7Y	G85	108 ± 10^o^	3 ± 7^o^	G85V
2	2LQB	G170	−74 ± 16^o^	−57 ± 3^o^	—
3	2ROP	G284	−98 ± 19^o^	−16 ± 22^o^	—
4	2ROP	G386	83 ± 11^o^	−15 ± 25^o^	—^1^
5	2EW9	G515	−72 ± 8^o^	−6 ± 19^o^	—
6	2EW9	G591	−75 ± 4^o^	−34 ± 6^o^	G591D

^1^Protein destabilization by G386V substitution observed *in vitro*.

In MBD1 and in MBD4, this residue adopts a backbone conformation that is only favorable for glycine, and is disallowed for valine: for G85 the backbone dihedral angle values are φ = 108 ± 10^o^ and ψ = 3 ± 7^o^, and, for G386, φ = 83 ± 11^o^ and ψ = −15 ± 25^o^. In the other MBDs, the corresponding glycine adopts backbone conformations falling into the universally allowed broad region of the Ramachandran plot surrounding the ideal α-helix values. Thus amino acid residues with bulky side chains will be disruptive at this position in MBD1 and MBD4, but not necessarily in the other MBDs. Analysis of the local dynamics is consistent with this interpretation. Residues 33–35 in MBD1, corresponding to K84-G85-I86 in the full-length ATP7B, all have high order parameter (*S*^2^) values over 0.98 indicating a rigid local structure, with very limited backbone flexibility (Fig. 1S,D).

Although, to the best of our knowledge, the G386V mutation in MBD4 has not been reported to cause Wilson disease, its effect has been tested *in vitro*. Consistent with the structural analysis, the G386V variant of MBD4 was shown to be unstable, with about 20^o^C lower midpoint temperature of thermal denaturation than the wild type protein^32^. The deleterious effect of G591D mutation in MBD6, where G591 has backbone dihedral angles of φ = −75 ± 4^o^ and ψ = −34 ± 6^o^, cannot be explained by the constraints of the backbone conformation, and likely has other causes, such as disruption of domain-domain interactions, or local effects caused by the negative charge of the aspartate side chain. In fact, MBD6 is the closest to the membrane and likely forms a larger number of interdomain contacts than any other MBD.

Misfolding of G85V-MBD1 may have further structural consequences for ATP7B in the cell. Our recent work indicates that MBD1-3 interact with each other, forming a dynamically correlated domain group^25^. These interactions may be involved in the regulation of ATP7B activity and trafficking by copper^24^. MBD1 misfolding will disrupt MBD1-3 interactions, and interfere with proper ATP7B trafficking and activity regulation. This, in turn may be followed by protein degradation, as reported previously.

In summary, the structure of MBD1 taken together with the previously solved structures of the other MBDs explains the disruptive effect of a Wilson disease causing mutation located in the regulatory region of the protein, where very few disease causing mutations have been reported.

## Methods

### Protein expression and purification

MBD1 was expressed as fusion with the chitin-binding domain and intein using vector pTYB12 (New England BioLabs). The protein was isotopically labeled with ^15^N and ^13^C, and purified by chitin affinity chromatography combined with intein self-cleavage, essentially as described previously^33^. Prior to NMR experiments, MBD1 was dialyzed against 50 mM HEPES pH 7.4, 50 mM NaCl, 5 mM *tris*-(2-carboxyethyl)phosphine.

### NMR experiments and structure determination

NMR experiments were performed on the 600 MHz and 750 MHz Bruker NMR spectrometers equipped with a CryoProbe. Data were processed using NMRPipe^34^ and analyzed using NMRView^35^. Backbone chemical shift assignments for MBD1 were made from HNCO, HNCA, HNCACB, HN(CA)CO, and HN(CO)CACB experiments^36^. Side-chain assignment experiments were done using H(CCO)NH, CC(O)NH, AND ^1^H, ^15^N-TOCSY. The backbone chemical shifts were assigned using CARA^37^. Hydrogen bond distance restraints were derived from H/D exchange measurements.

Peak tables and distance restraint generation from 3D ^1^H, ^15^N-NOESY and 3D ^1^H, ^13^C-NOESY experiments, as well structure calculation was done using PONDEROSA^38^. Peak tables and distance restraint generation from 3D ^1^H, ^15^N-NOESY and 3D ^1^H, ^13^C-NOESY experiments, as well structure calculation was done using PONDEROSA-C/S^38^ with XPLOR-NIH based calculation options^39^. NOE cross peaks were automatically assigned with AUDANA^40^ and manually verified by PONDEROSA Analyzer coupled with NMRFAM-SPARKY^41^ and PyMOL (Schroedinger LLC). Final structure calculation step was carried out with explicit water refinement option with validated restraints that selects best 20 from 100 calculated models with the lowest energy criteria. The structure has been deposited in RCSB as entry 2N7Y.

NMR relaxation measurements, and data analysis using TENSOR 2.0^42^ were performed essentially as described previously^25^, except that HSQC rather than TROSY versions of NMR experiments were used.

## Electronic supplementary material

Supplementary material
