# Supplementary material for: The Structure of Metal Binding Domain 1 of the Copper Transporter ATP7B Reveals Mechanism of a Singular Wilson Disease Mutation
Source: Sci Rep. 2018 Jan 12;8:581. doi: 10.1038/s41598-017-18951-1 (PMC5766562; doi:10.1038/s41598-017-18951-1)
Supplement: Supplementary file 1 — Supplementary material [file 41598_2017_18951_MOESM1_ESM.pdf]

**The Structure of Metal Binding Domain 1 of the Copper Transporter ATP7B Reveals Mechanism of a Singular Wilson Disease Mutation.**

**Corey H. Yu, Woonghee Lee, Sergiy Nokhrin and Oleg Y. Dmitriev**

## Supplementary information

**Table S1:** Statistics for structure calculation of MBD1.

| Parameter | Value |
|---|---|
| Total number of NOE distance restraints | 628 |
| Intra-residue | 305 |
| Sequential (\|i – j\| = 1) | 143 |
| Medium range (\|i – j\| < 5) | 49 |
| Long range (\|i – j\| > = 5) | 91 |
| Hydrogen bond restraints | 40 |
| Dihedral angle constraints | 119 |
| Phi (ϕ) | 54 |
| Psi (ψ) | 65 |
| RMSD for bond angles (°) | 0.6 |
| RMSD for bond lengths (Å) | 0.008 |
| RMSD from experimental restraints | |
| NOE (Å) | 0.016 ± 0.002 |
| Dihedral angles (°) | 0.452 ± 0.008 |
| Mean NOE violations larger than 0.25 Å | 0 |
| Maximum NOE violation (Å) | 0.27 |
| Average RMSD to the mean (Å) | |
| Backbone | 0.59±0.16 |
| All heavy atoms | 1.15±0.23 |
| PROCHECK Z-scores (ϕ an ψ/all dihedral angles) | 1.14/1.12 |
| MolProbity clash score/Z-score | 13.88/-0.86 |
| Ramachandran plot summary from PROCHECK (%) | |
| Residues in most favorable regions | 97.3 |
| Residues in additionally allowed regions | 2.7 |
| Residues in disallowed regions | 0.0 |
| Ramachandran plot summary from MolProbity (%) | |
| Residues in most favorable regions | 99.4 |
| Residues in additionally allowed regions | 0.6 |
| Residues in disallowed regions | 0.0 |
| Average number of angle constraint violations per conformer (>5°) | 0 |

**Fig. S1**

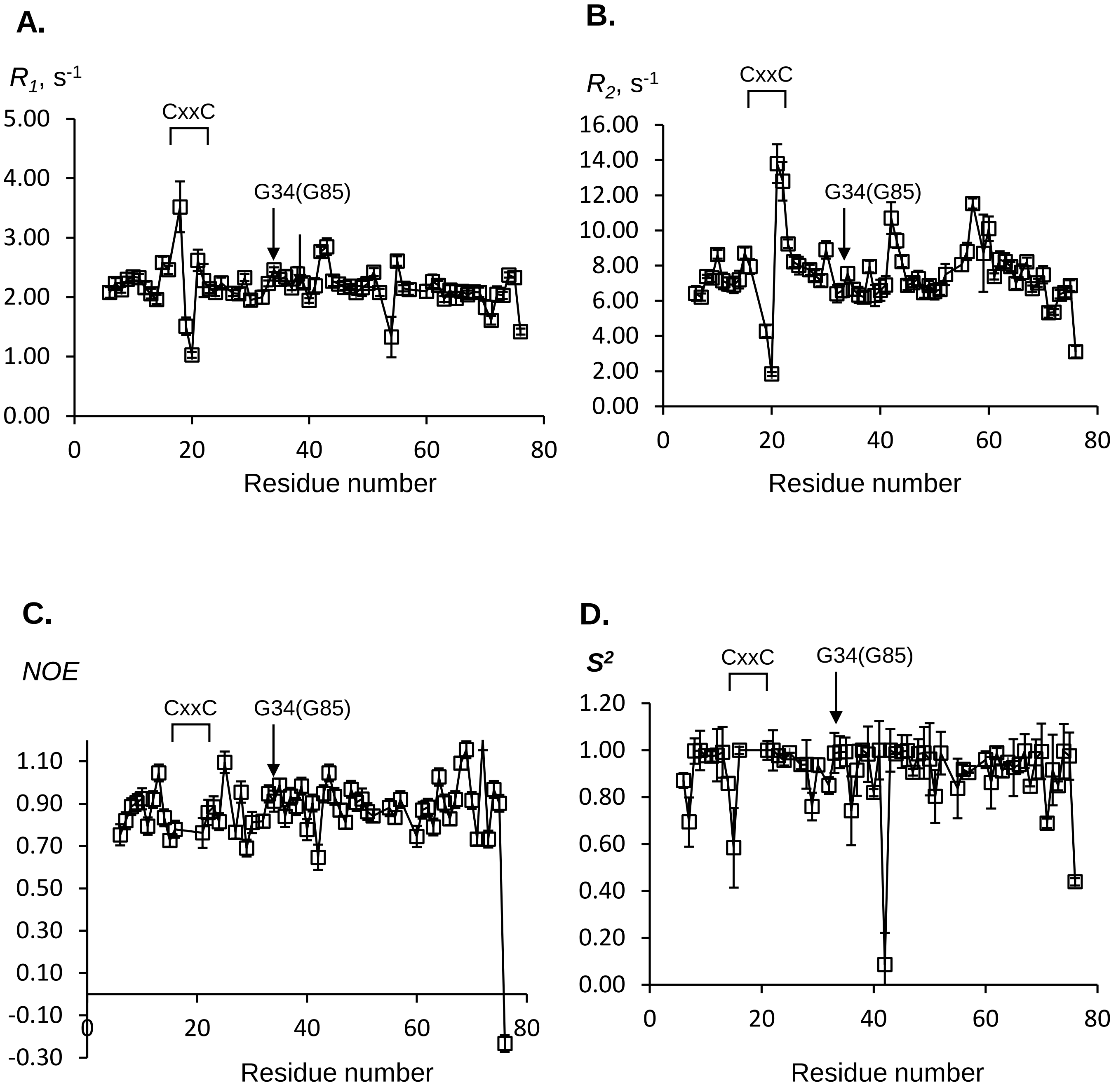


**MBD1 dynamics.** Relaxation rates $R_1$ (**A**) and $R_2$ (**B**), heteronuclear $^{15}N$-{$^1H$} NOEs (**C**), and order parameter $S^2$ (**D**) plotted as functions of residue number. Data points for G34, corresponding to G85 in the full-length ATP7B are marked with an arrow, and data points for the residues in the conserved copper binding motif (C18 to C21 in MBD1, corresponding to C69 to C72 in the full length ATP7B) with a square bracket.
